# Emerging of Cutaneous Leishmaniais Due to *Leishmania major* in a New Focus in Esfahan Province, Central Iran

**DOI:** 10.18502/jad.v14i2.3731

**Published:** 2020-06-30

**Authors:** Reza Jafari, Hamid Abdoli, Mohammad Hossein Arandian, Nilofar Shareghi, Maryam Ghanei, Nilofar Jalali-Zand, Shahram Nekoeian, Arshad Veysi, Ahmad Montazeri, Amirabdollah Ghasemi, Javad Ramazanpour, Reza Fadaei, Amir Ahmad Akhavan

**Affiliations:** 1 Esfahan Health Research Station, School of Public Health, Tehran University of Medical Sciences, Esfahan, Iran; 2 Isfahan Province Health Center, Isfahan University of Medical Sciences, Isfahan, Iran; 3 Zoonoses Research Center, Research Institute for Health Development, Kurdistan University of Medical Sciences, Sanandaj, Iran; 4 Naein Health Care Network, Isfahan University of Medical Sciences, Isfahan, Iran; 5 Department of Medical Entomology and Vector Control, School of Public Health, Tehran University of Medical Sciences, Tehran, Iran

**Keywords:** Cutaneous leishmaniais, *Leishmania major*, Epidemiology, Central Iran

## Abstract

**Background:**

Esfahan Province is considered as one of the main focus of zoonotic cutaneous leishmaniasis (ZCL) in Iran. Recently, ZCL distribution is expanding through this province leading to report of new cases in non-endemic areas. In the current study epidemiological aspects of ZCL has been investigated in Naein County in Esfahan Province.

**Methods:**

Adult sand flies were collected from beginning to the end of their seasonal activity. Rodents were caught by Sherman live traps once a month for one year. To active case detection, a hundred households in each selected village were visited in November and December 2016. Nested-PCR was employed to detect *Leishmania* parasite in the vector, reservoir and human.

**Results:**

Totally 1562 sand flies including *Phlebotomus sergenti*, *Phlebotomus papatasi*, *Sergentomyia sintoni* and *Sergentomyia mervinae* were collected and identified. No *Leishmania* infection was detected in the collected sand flies. All of the 30 collected rodents were identified as *Rhombomys opimus*, and of these 3.3% and 26.7% were infected by *Leishmania major* using microscopic and molecular technique respectively. Totally, 914 individuals were investigated and the ulcer and scar rates of ZCL calculated to be at 1.1 and 15.3 per 1000 population, respectively. Molecular results confirmed *L. major* infection in human and reservoir samples.

**Conclusion:**

It is concluded that ZCL is established in the area in low endemicity, and it is extrapolated the disease will not be a serious increasing health problem in the near future in this region.

## Introduction

Although estimated to cause ninth largest disease burden among infectious diseases, leishmaniasis is mostly neglected among tropical diseases. Based on a recent report, it is estimated that, annually 0.2 to 0.4 and 0.7 to 1.2 million visceral leishmaniasis (VL) and cautaneus leishmaniasis (CL) new cases, occur respectively in the world (1). High level of reported cases listed Iran in ten countries which together account for 70 to 75% estimated cases of CL globally (1). More than 20000 cases of CL cases are reported in Iran annually, although the actual figures are estimated to be 4 to 5 folds (2). Cutaneous leishmaniasis manifest in two forms in the country, zoonotic (ZCL) and anthroponotic CL (ACL), and about 80% of reported cases has been ZCL form; especially in the rural areas of the country (3). Zoonotic cutaneous leishmaniasis is caused by a protozoan parasite *Leismania major*, and *Phlebotomus papatasi* plays as the main vector in the disease foci inside the country. Seventeen out of 31 province deal with the disease (4). Rodents belonging to Gerbillinae subfamily play as reservoir host of ZCL in different part of the country, as well as some other parts of the world. *Rhombomys opimus* (great gerbil) known as the main reservoir host of ZCL in central and the northeast of Iran (5). Esfahan Province known as one of the most important foci of ZCL, and in 2012 this province was categorized as one of the high reported cases of ZCL (6). Since the last two decades, Iranian scientists along with health authorities have employed and conducted several measures such as spraying of rodent burrows with pesticides (7), baiting the reservoir hosts with rodenticides (8–13) and using deltamethrin-impregnated bed nets and curtains (14, 15) to control ZCL in the country. In addition, despite extensive research in the field of vaccine, still there is no licensed vaccine against any form of leishmaniasis (16). Iranian researchers have conducted a successful leishmanisis prevention through leishmanization, unfortunately it was banned due to very rare case of un-healing lesions, and since then it was recommended just for military personnel in the high risk areas (17). From 2012 in Naein County the number of indigenous cases which were visited in the Health Centers with signs of cutaneous leishmaniais gradually increased and based on local health authorities report, surprisingly the confirmed recorded cases increased to 26 cases in 2015. Considering the importance of this county as a main roads intersection of the country and passengers commuting, it is assumed, without taking serious measures to conduct research and control of the disease in this region, it might become a potential focus to spread ZCL to other free areas. Regarding this issue as well as lacking any comprehensive study on ZCL in this county, conducting an epidemiological research seemed to be necessary. Hence, this survey is the first epidemiological study that has ever been conducted in these areas. The current study aimed to study the different epidemiological aspects of the disease in this new emerging focus of ZCL.

## Materials and Methods

### Study area

**Fig. 1.**
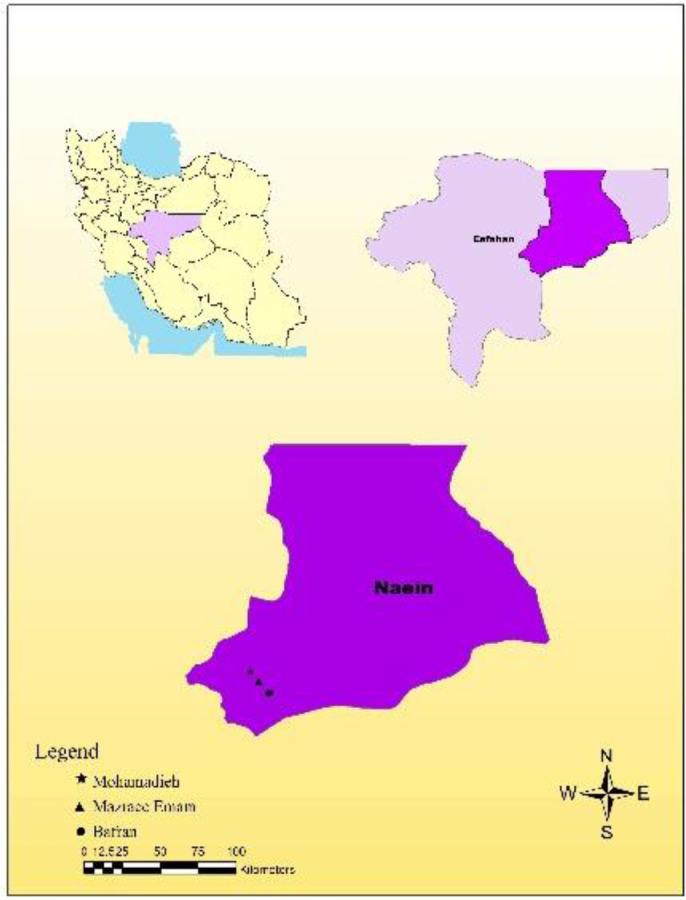
Map of the studied areas, Naein County, Esfahan Province, Iran

The present study was carried out in Naein County (32°51’49.1”N 53°05’04.5”E), Esfahan Province, Iran from October 2015 to February 2016. Naein is located at 170km from north of Yazd and 140km from east of Esfahan with an area about 22,570km^2^ and it is considered as the widest County in Esfahan Province (Fig. 1).

The selected areas have desert climate, hot in summer and cold in winter. In 2015 the maximum and minimum mean monthly temperature was 40.8 and −10.6 °C in July and December, respectively; with the total rainfall of 40.8mm.

### Entomological survey

Adult sand flies were collected from the beginning (April) to the end (November) of their active season, once a month using sticky traps in the rural selected areas (Bafran, Mohamadieh and Mazraee Emam) of Naein. The sticky traps (15 in outdoor resting places and 15 in indoors in each selected point) were installed before sunset and collected next morning before sunrise in three selected villages. The captured sand flies were preserved in ethanol until the time of use. Head and the last two abdomen segments of sand flies detached, mounted in Puri’s media and the rest of the sand flies’ body was kept in 96% alcohol to examine for *Leishmania* infection using molecular techniques. The species identified using valid keys based on morphological characters (18, 19).

### Rodent collection and parasitological examination

Active colonies of gerbils have been found around Bafran, Mohamadieh and Mazraee Emam villages of Naein. Rodents were caught by 40 Sherman live traps, baited with cucumber and/or carrot, once a month along a year. During spring to fall, the traps were installed in the vicinity of the gerbil holes in the afternoon and collected at the following morning, while during winter traps were installed at early mornings and collected at the afternoons. The collected gerbils were transferred to the Esfahan Health Research Station to further examination. The captured rodents were anaesthetized using Ketamine hydrochloride (60mg/kg), and Xylazine (5mg/kg) and were identified by morphological characters (20). Then impression smears were prepared from the ear lobes of the anaesthetized animals, fixed by absolute methanol and stained by Giemsa, and then the slides were microscopically examined at high magnification (1,000x). Afterward the ear lobes were cut and transferred into 500μl cold PBS (pH= 7.4), and then disrupted by grinding using a pestle and stored in −20 °C until use.

Animal ethics was considered in treating with animals during this study.

### Human infection

To determine the prevalence and incidence of the disease in the selected village areas (Bafran, Mohamadieh and Mazraee Emam) of Naein, 100 households in each village were visited in November and December 2016. Epidemiologic data including ID, presence or absence of scar (s) or active lesion (s), number of the lesion (s) or scar (s), and history of travelling to the other ZCL foci were filled out in a questionnaire for all households. Individuals who had travelled to other foci of ZCL were excluded from the study. New cases of the disease and the number of active lesions were recorded on each visit. Samples were prepared from the active lesions; before sampling, lesions were disinfected with alcohol 70% then the margin of ulcer was scratched using vaccinostyle. The prepared serosity was allocated in two parts, one part transferred into alcohol 96% for molecular parasite detection and another part was smeared on a slide, fixed with methanol, stained by Giemsa and examined under microscope at high magnification (1,000x).

### Molecular detection of *Leishmania* infection

Genomic DNA from samples of rodents, human and sand flies was extracted by GeneAll® Exgene TM Tissue kit (Cat No: 101–109) following the protocol of cultured animal cell or lymphocytes. The ITS2 region of *Leishmania* parasite was amplified by Nested-PCR, using the following primers (21) leish out F (5′-AAA CTC CTC TCT GGT GCT TGC-3′), Leish out R (5′-AAA CAA AGG TTG TCG GGG G-3′), Leish in F (5′-AAT TCA ACT TCG CGT TGG CC-3′) and Leish in R (5′-CCT CTCTTT TTT CTC TGT GC-3′). The targeted gene of ribosomal DNA was amplified by a Biosystems thermocycler. The volume of first reaction micro-tube of PCR was 25μl containing 0.6μM of both forward (Leish out F) and reverse (Leish out R) external primers, 12.5μl Taq DNA polymerase enzyme, 2X Master Mix (Amplicon, Denmark) and sterile distilled water. The first denaturation step was carried out at 95 °C for 5min and was followed by 30 cycles of denaturation at 94 °C for 30s, annealing at 60 °C for 45s and extension at 72 °C for 1min, finally ended with a final extension step of 72 °C for 5min. The second step of nested-PCR was carried out in a volume of 20μl containing 1μl of a 1:25 dilution of distilled water and the first-round PCR product as a template, 0.3μM of each forward and reverse internal primers, 10μl of Taq DNA polymerase and 2X Master mix. The amplification was performed as initial denaturation at 95 °C for 2min, 25 cycles of 94 °C for 15s, 62 °C for 30s, 72 °C for 45s followed by the final extension at 72 °C for 5min. Finally, PCR products were loaded on 1.5% (w/v) agarose gel electrophoresis in TBE buffer (0.09mM Tris, 0.09mM boric acid, and 20mM EDTA, pH= 8.3); ethidium bromide (0.5μg/ml) was used to visualize the amplified DNA bands on the gel and photographs were taken.

*Leishmania major* (MRHO/IR/75/ER), and distilled water were used as positive and negative controls respectively. To prevent any accidental contamination, primary precautions such as using filter pipette tips, and sterilizing equipment by 10% sodium hypochlorite solution were taken.

### Polymerase chain reaction-restriction fragment length polymorphism (PCRRFLP)

To distinguish common species of *Leishmania* genus, the products of Nested-PCR were subjected to RFLP using Rapid Digest Mnl1 (Cat. No: RD 1191) restriction enzyme. To this purpose, 10μl PCR products of the nested-PCR, 2μl Buffer, 1μl Mnl1 restriction enzyme, were added to a micro tube and reached to final volume of 30μl by distilled water. To activate the restriction enzyme, the mixture was kept in 37 °C for 1 hour, and then the products were loaded onto 2.5% (w/v) agarose gel electrophoresis in TBE buffer. Finally loaded DNA was visualized by ethidiumbromide (0.5μg/ml), and photographed.

## Results

### Entomological survey

**Fig. 2.**
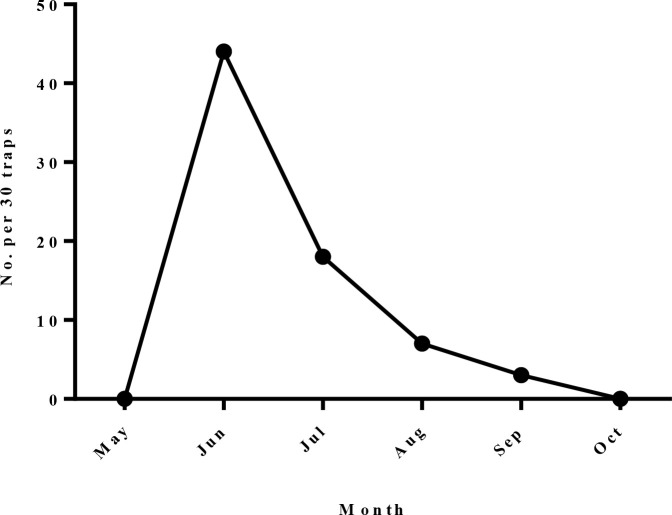
Monthly fluctuation of *Phlebotomus sergenti* in indoors of the studied areas, Naein County, Esfahan Province, Iran, 2011

From April to November 2016, totally 1562 sand flies (1451 from outdoors, 111 from indoors) were collected and identified based on the valid morphological keys. Four species of sand flies including two species of *Phlebotomus* genus and two species of *Sergentomyia* genus were identified as follows:

***Phlebotomus (Paraphlebotomus) sergenti:*** This sand fly was the dominant species in indoors, which 70 (63.1%) and 8 (0.6%) of collected sand flies in indoors and outdoors were belonged to this species respectively. *Phlebotomus sergenti* was caught in all months of sand flies active season in indoor places. The maximum relative frequency of this phlebotomine in indoor places was recorded in June (Fig. 2).

**Fig. 3.**
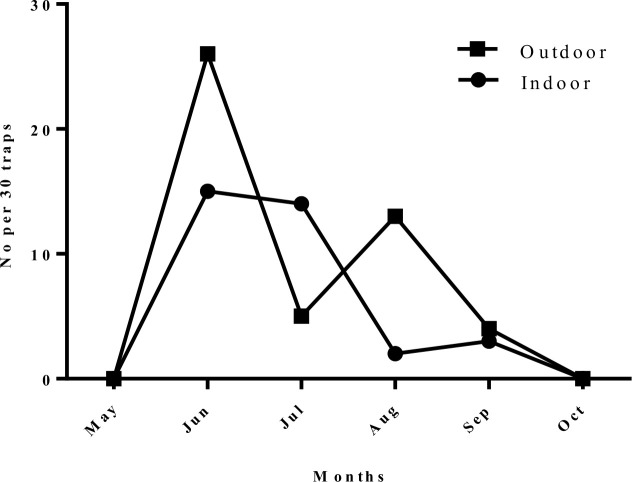
Monthly fluctuation of *Phlebotomus papatasi* in studied areas, Naein County, Esfahan Province, Iran, 2011

***Phlebotomus* (*Phlebotomus*) *papatasi*:** The number of caught sand flies belonging to this species were 34 (30.6%) and 48 (3.3%) in indoors and outdoors respectively. *Phlebotomus papatasi* was caught in all months of sand flies active season in either indoors or outdoors. The monthly activity of this species recorded a remarkable pick in June, both in indoor and outdoor places (Fig. 3).

**Fig. 4.**
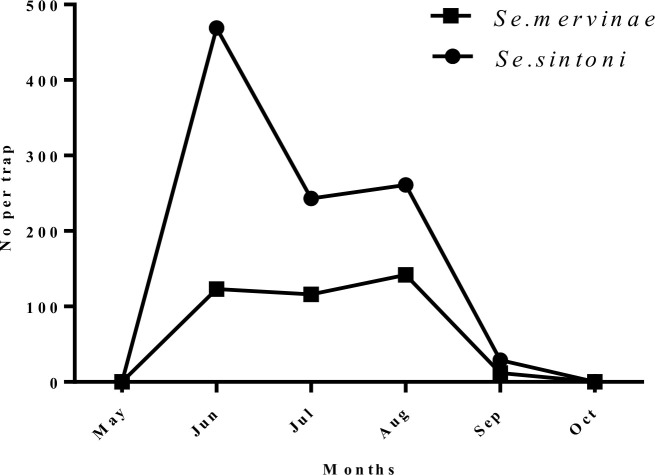
Monthly fluctuation of *Sergentomyia sintoni* and *Sergentomyia mervinae* in outdoors of the studied areas, Neain County, Esfahan Province, Iran, 2011

***Sergentomyia* (*Sergentomyia*) *sintoni*:** The results showed *Se. Sintoni* was the most dominant species in outdoor places. Of all collected sand flies, 4 (3.6%) from indoor and 1002 (69%) from outdoors places were identified as *Se. sintoni*. Similarly, the maximum activity of this species occurred in June (Fig. 4).

***Sergentomyia* (*Sergentomyia*) *mervinae*:** This species comprised 3 (2.7%) of the sand flies from indoors, and 393 (27.1%) from outdoor resting places. Similarly, the maximum number of this species was caught in June (Fig. 4).

To find *Leishmania* infection among *Ph. papatasi* randomly 30 females were examined by nested-PCR technique. Results of molecular experiment showed none of the sand flies were infected by *Leishmania* parasite.

Sufficient numbers of *Ph. sergenti* from outdoors and *Se. sintoni* and *Se. mervinae* from indoors were not collected to demonstrate monthly activity.

### Reservoir host investigation

**Fig. 5.**
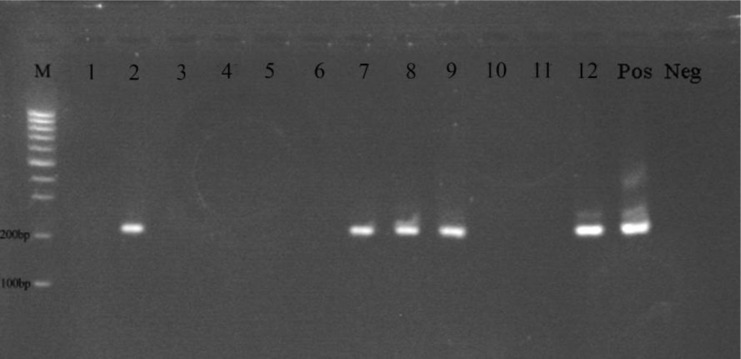
Gel electrophoresis of nested-PCR targeted sequences of DNA extracted from the smears of the captured rodents. M; 100bp DNA ladder (Fermentas), 2, 7, 8, 9 and 12 showing the samples were positive for *Leishmania major.* Pos; positive *L. major* reference. Neg; negative control

Totally 30 desert rodent were collected by Sherman live-tarps during a year, and all identified as *R. opimus* based on the morphological characteristics. Out of 30 examined rodents, only one (3.3%) was infected by amastigote form of *Leishmania* through microscopic examination. In addition, by using molecular examination eight (26.7%) of the captured rodents were found to be infected by *L. major* (Fig. 5).

### Human infection survey

**Fig. 6.**
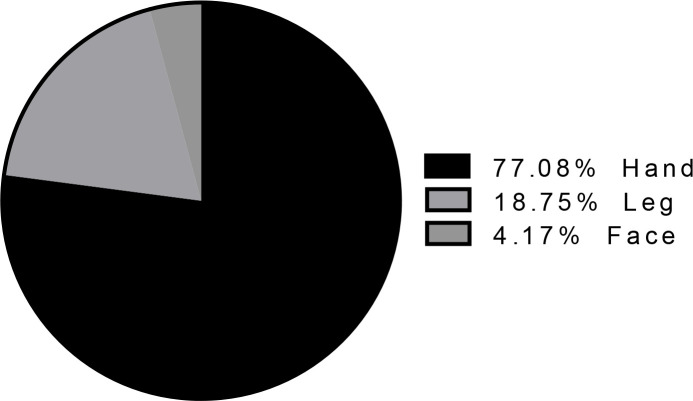
The percent of scars on the body of visited inhabitant in the studied areas, Neain County, Esfahan Province, Iran, 2011

To determine scar rate and ulcer rate of ZCL among inhabitants, active case finding was carried out at the end of fall in Mohamadieh, Bafran and Mazraee Emam villages. Taken together, 914 individuals including 449 males (49.1%) and 465 females (50.9%) were investigated and demographic data were recorded as well. Among all visited individuals only one woman (0.1%) in ≥25 age-group had one active lesion of ZCL on her hand. Out of 914 examined persons, 14 individuals (1.5%) had scar which eight (1.8%) were man and six (1.3%) were woman belonging to ≥ 25 age-group. The distribution rate of scar site/s on the body of examined people is shown in Fig. 6.

The ulcer and scar rates of ZCL calculated to be at 1.1 and 15.3per 1000 population, respectively (Table 1). Molecular experiments on one sample of active lesion confirmed *L. major* infection.

**Table 1.** The prevalence of scar rate of the examined population in the studied villages, Naein County, Esfahan Province, Iran, 2011

**Age groups (yr)**	**Male**			**Female**			**Total**		

	**No. observed**	**No. of scar/s**		**No. observed**	**No. of scar/s**		**No. observed**	**No. of scar/s**	
		
		**No**	**%**		**No**	**%**		**No**	**%**
**0–4**	22	0	0	25	0	0	47	0	0
**5–9**	29	0	0	24	0	0	53	0	0
**10–14**	25	0	0	26	0	0	51	0	0
**15–19**	26	0	0	25	0	0	51	0	0
**20–24**	49	0	0	42	0	0	91	0	0
**≥ 25**	298	8	2.7	323	6	1.9	621	14	2.3
**Total**	449	8	1.8	465	6	1.3	914	14	1.5

## Discussion

The current study is the first epidemiological survey on zoonotic cutaneous leishmaniasis in Naein County, Esfahan Province. In the current study four species of sand flies including two species of *Phlebotomus* and two species of S*ergentomiya* genus were collected and identified. In indoor places *Ph. sergenti* with the 63.1% density was the most abundant species and *Ph. papatasi* with 30.6 % density ranked in the second place. These two species were caught throughout the sand flies’ active season. Generally, *Sergentomyia* species prefer to feed on cold-blooded vertebrates, thus they are known as vectors of reptile *Leishmania* species. It is widely believed, they cannot transmit *Leishmania* to human, although promastigote infection of *Se. sintoni* have shown by direct examination previously (22). However, they are usually occurred sympatrically with *Phlebotomus* species, even though in some cases, same to this study, they have higher density comparing to *Phlebotomus* genus. Hence, the role of these sand flies in transmitting of human leishmaniaisis needs more investigation. Majority of studies conducted in central Iran have shown *Ph. papatasi* as the predominant species in either indoors and outdoors places (23, 24). In agreement with our result, generally it has been shown there were two peaks in the density curve of the most species in the hot and arid areas of central part of the country; one in June or July and the second in August or September (23, 24). Of 30 *Ph. papatasi* were passed to nested-PCR experiment none of them were infected by *Leishmania* parasite. Natuaral *Leishmania* infection has been reported from *Ph. papatasi*, *Phlebotomus caucasicus*, *Phlebotomus ansarii* and *Se. sintoni* (22–24), and also *L. major* was detected in *Ph. papatasi* using molecular methods (25–27).

In the current study out of 30 captured *R. opimus*, 8 (26.7%) were infected by *L. major* using nested-PCR, and reconfirmed by PCRRFLP technique. Till now, several studies have conducted in the country showed *Leishmania* infection in *R. opimus* by either direct examination or molecular methods (5, 28–30). As the results showed, the detected *Leishmania* species from *R. opimus* and human lesion was the same. In the previous studies *L. major* has been isolated from naturally infected *Ph. papatasi*, *Ph. caucasicus*, *R. opimus*, *Meriones libycus* and human in endemic area of ZCL in the country (5).

Ulcer and scar rates among surveyed population were 0.1% and 1.5% respectively, these rates were reported as 1.3% and 3.26% in other county of Esfahan Province (31). The most infected age-group was ≥ 25, while in the previous study was 10–14 age-group (31). In an epidemic situation, the rates of ulcer and scar were reported as 3% and 10.4% respectively, and also the most infected age-group was 0–4 years old (22).

This finding showed high level of endophilic behavior for *Ph. sergenti*. It is worth to mention that high density of *Ph. sergenti*, the main vector of ACL, in indoors companioning to a case of ACL or dogs infected by *L. tropica* in the areas, pose a potential threat of ACL establishment in the region. On the other hand, despite the high density of *Ph. sergenti* and its refractory to *L. major*, so it cannot play a role in transmitting of *L. major* in the areas. Considering the results of the current study, this area is not categorized as a high risk area for ZCL transmission due to *L. major*. Due to low density and no *Leishmania* infection in potential vector of ZCL, low population of reservoir host, sporadic cases and lack of history of the disease below 25 years old group, this area could be considered as a low risk focus.

## Conclusion

It is concluded that ZCL has established in low endemicity in this area. It is extrapolated that, the disease will not be a serious health problem in the near future in this region. Based on the results of this study, presently no vector and/or reservoir host control is recommended. Only treatment of rare new cases with active lesion following the national protocol is recommended. Considering climate changes and ecological status, consequent probable epidemiological change of ZCL is not unexpected, so that continuing of disease surveillance to take urgent action in special condition is recommended.
